# Supplementary material for: No evidence for negative impacts of acute sulfoxaflor exposure on bee olfactory conditioning or working memory
Source: PeerJ. 2019 Aug 12;7:e7208. doi: 10.7717/peerj.7208 (PMC6694785; doi:10.7717/peerj.7208)
Supplement: Table S1 [file peerj-07-7208-s001.pdf]

Table S1: Table of candiate models used for each analysis.

A) Bumblebee PER, binomial learning; analysed with a generalized linear mixed effect model (binomial); colony included as random factor	AICc	ΔAICc	*w* i
**Null model**	**129.7**	**0**	**0.658**
**Bee size**	**131.7**	**1.94**	**0.249**
**Treatment**	**134.3**	**4.6**	**0.066**
Treatment + Bee size	136.2	6.5	0.026
Treatment + Bee size + Treatment:Bee size	142.4	12.65	0.001
**B) Honeybee PER, binomial learning; analysed with a generalized linear mixed effect model (binomial); colony included as random factor**	AICc	ΔAICc	*w* i
**Null model**	**134.4**	**0**	**0.54**
**Bee size**	**136.5**	**2.1**	**0.189**
**Treatment**	**137.3**	**2.91**	**0.126**
**Treatment + Bee size + Treatment:Bee size**	**137.7**	**3.34**	**0.102**
Treatment + Bee size	139.4	5.06	0.043
**C) Bumblebee PER, learning level; analysed with a generalized linear mixed effect model (poisson); colony included as random factor**	AICc	ΔAICc	*w* i
**Null model**	**215.8**	**0**	**0.533**
**Bee size**	**216.2**	**0.4**	**0.437**
Treatment	222.7	6.85	0.017
Treatment + Bee size	223.3	7.47	0.013
Treatment + Bee size + Treatment:Bee size	229.9	14.12	0
**D) Honeybee PER, learning level; analysed with a generalized linear mixed effect model (poisson); colony included as random factor**	**AICc**	**ΔAICc**	***w* i**
**Bee size**	**284**	**0**	**0.492**
**Null model**	**285.1**	**1.11**	**0.283**
**Treatment + Bee size**	**287.6**	**3.61**	**0.081**
**Treatment + Bee size + Treatment:Bee size**	**287.7**	**3.66**	**0.079**
**Treatment**	**288.1**	**4.06**	**0.065**
**E) Bumblebee PER, Speed to learn**	**AICc**	**ΔAICc**	***w* i**
**Null model**	**413.3**	**0**	**0.587**
**Bee size**	**414.6**	**1.28**	**0.309**
**Treatment**	**417.8**	**4.46**	**0.063**
Treatment + Bee size	418.8	5.5	0.038
Treatment + Bee size + Treatment:Bee size	423.2	9.9	0.004
**F) Honeybee PER, Speed to learn**	**AICc**	**ΔAICc**	***w* i**
**Null model**	**391.7**	**0**	**0.579**
**Bee size**	**393.7**	**2.05**	**0.208**
**Treatment**	**394.6**	**2.94**	**0.133**
Treatment + Bee size	396.8	5.11	0.045
Treatment + Bee size + Treatment:Bee size	397.3	5.66	0.034
**G) Bumblebee PER, Memory 3H**	**AICc**	**ΔAICc**	***w* i**
**Null model**	**58.9**	**0**	**0.64**
**Bee size**	**60.8**	**1.87**	**0.251**
**Treatment**	**63.2**	**4.27**	**0.075**
Treatment + Bee size	65	6.13	0.03
Treatment + Bee size + Treatment:Bee size	69.1	10.25	0.004
**H) Honeybee PER, Memory 3H**	**AICc**	**ΔAICc**	***w* i**
**Null model**	**101.5**	**0**	**0.526**
**Bee size**	**102**	**0.47**	**0.416**
Treatment	107.1	5.55	0.033
Treatment + Bee size	107.8	6.22	0.023
Treatment + Bee size + Treatment:Bee size	113.8	12.27	0.001
**I) Bumblebee PER, Memory 24H**	**AICc**	**ΔAICc**	***w* i**
**Null model**	**50.9**	**0**	**0.716**
**Bee size**	**53.2**	**2.27**	**0.23**
Treatment	56.6	5.65	0.042
Treatment + Bee size	59.3	8.36	0.011
Treatment + Bee size +Treatment:Bee size	67.3	16.32	0
**J) Honeybee PER, Memory 24H**	**AICc**	**ΔAICc**	***w* i**
**Null model**	**96.5**	**0**	**0.453**
**Bee size**	**97.9**	**1.4**	**0.225**
**Treatment**	**98**	**1.43**	**0.222**
**Treatment + Bee size**	**99.6**	**3.08**	**0.097**
Treatment + Bee size +Treatment:Bee size	106.5	9.94	0.003
**K) RAM, total revisits**	**AICc**	**ΔAICc**	***w* i**
**Null model**	**321.7**	**0**	**0.468**
**Treatment**	**323**	**1.37**	**0.236**
**Bee size**	**323.2**	**1.51**	**0.22**
**Treatment + Bee size**	**325.4**	**3.75**	**0.072**
Treatment + Bee size + Treatment:Bee size	330.5	8.8	0.006
**L) RAM , correct in first 8**	**AICc**	**ΔAICc**	***w* i**
**Null model**	**229**	**0**	**0.706**
**Bee size**	**231.1**	**2.13**	**0.244**
Treatment	234.8	5.84	0.038
Treatment + Bee size	237.2	8.23	0.012
Treatment + Size + Treatment:Bee size	244.7	15.74	0
**M) RAM , correct before revisit**	**AICc**	**ΔAICc**	***w* i**
**Treatment**	**356.6**	**0**	**0.437**
**Null model**	**357.3**	**0.72**	**0.306**
**Treatment + Bee size**	**358.9**	**2.27**	**0.141**
**Bee size**	**359.4**	**2.76**	**0.11**
Treatment + Bee size + Treatment:Bee size	365.2	8.57	0.006
