# Supplementary material for: No evidence for negative impacts of acute sulfoxaflor exposure on bee olfactory conditioning or working memory
Source: PeerJ. 2019 Aug 12;7:e7208. doi: 10.7717/peerj.7208 (PMC6694785; doi:10.7717/peerj.7208)
Supplement: Table S2 — Parameters highlighted in bold have 95% confidence intervals that do not cross zero [file peerj-07-7208-s002.pdf]

Table S2. Parameter estimates and 95% confidence intervals derived by model averaging across the confidence set of models. Parameters highlighted in bold have 95% confidence intervals that do not cross zero.

A) Bumblebee PER, binomial learning	Estimate	Std. Error	Lower CI	Upper CI
Intercept	-0.50	1.65	-3.78	2.77
Bee size	0.06	0.31	-0.55	0.68
Treatment (2.4 ppb)	0.00	0.17	-0.34	0.33
Treatment (10 ppb)	0.00	0.18	-0.35	0.36
Treatment (250 ppb)	0.05	0.24	-0.43	0.53
B) Honeybee PER, binomial learning	Estimate	Std. Error	Lower CI	Upper CI
(Intercept)	1.06	6.13	-11.06	13.19
Bee size	-0.27	1.57	-3.38	2.84
Treatment (2.4 ppb)	-1.30	6.51	-14.19	11.60
Treatment (10 ppb)	-1.26	6.85	-14.82	12.31
Treatment (250 ppb)	-7.32	23.30	-53.10	38.45
Bee size:Treatment (2.4 ppb)	0.28	1.64	-2.98	3.53
Bee size:Treatment (10 ppb)	0.31	1.76	-3.18	3.81
Bee size:Treatment (250 ppb)	1.91	6.05	-9.98	13.79
C) Bumblebee PER, learning learning	Estimate	Std. Error	Lower CI	Upper CI
(Intercept)	0.37	1.11	-1.84	2.57
Bee size	0.14	0.21	-0.29	0.56
D) Honeybee PER, learning learning	Estimate	Std. Error	Lower CI	Upper CI
(Intercept)	4.46	3.89	-3.25	12.18
Bee size	-2.37	2.67	-7.66	2.92
Treatment (2.4 ppb)	1.18	4.77	-8.23	10.59
Treatment (10 ppb)	1.05	4.05	-6.93	9.04
Treatment (250 ppb)	0.31	2.20	-4.11	4.72
Bee size	0.04	0.28	-0.53	0.61
Bee size:Treatment (2.4 ppb)	-0.33	1.26	-2.81	2.15
Bee size:Treatment (10 ppb)	-0.29	1.08	-2.42	1.83
Bee size:Treatment (250 ppb)	-0.09	0.57	-1.23	1.06
E) Bumblebee PER, Speed to learn	Estimate	Std. Error	Lower CI	Upper CI
Bee size	0.11	0.28	-0.41	1.12
Treatment (2.4 ppb)	0.00	0.11	-0.93	0.78
Treatment (10 ppb)	0.00	0.12	-0.91	0.87
Treatment (250 ppb)	0.03	0.15	-0.39	1.22
F) Honeybee PER, Speed to learn	Estimate	Std. Error	Lower CI	Upper CI
Bee size	0.01	0.56	-1.09	1.12
Treatment (2.4 ppb)	-0.11	0.31	-0.72	0.51
Treatment (10 ppb)	-0.02	0.16	-0.34	0.29
Treatment (250 ppb)	-0.01	0.15	-0.30	0.28
G) Bumblebee PER, Memory 3H	Estimate	Std. Error	Lower CI	Upper CI
(Intercept)	-2.38	3.14	-8.65	3.90
Bee size	0.16	0.58	-1.00	1.33
Treatment (2.4 ppb)	0.02	0.30	-0.59	0.63
Treatment (10 ppb)	-0.07	0.45	-0.98	0.83
Treatment (250 ppb)	0.06	0.34	-0.62	0.75
H) Honeybee PER, Memory 3H	Estimate	Std. Error	Lower CI	Upper CI
(Intercept)	-5.93	7.58	-20.89	9.03
Bee size	2.71	2.13	-2.65	5.05
I) Bumblebee PER, Memory 24H	Estimate	Std. Error	Lower CI	Upper CI
(Intercept)	-1.45	2.42	-6.34	3.45
Bee size	0.05	0.48	-0.92	1.02
J) Honeybee PER, Memory 24H	Estimate	Std. Error	Lower CI	Upper CI
(Intercept)	2.70	5.76	-8.72	14.12
Bee size	-0.61	1.49	-3.57	2.36
Treatment (2.4 ppb)	-0.39	0.71	-1.79	1.02
Treatment (10 ppb)	-0.36	0.66	-1.66	0.94
Treatment (250 ppb)	0.04	0.42	-0.79	0.88
K) RAM, total revisits	Estimate	Std. Error	Lower CI	Upper CI
(Intercept)	1.96	1.43	-0.88	4.79
Treatment (5 ppb)	0.24	0.41	-0.57	1.05
Treatment (10 ppb)	0.16	0.31	-0.46	0.79
Treatment (250 ppb)	0.23	0.39	-0.55	1.00
Bee size	-0.08	0.23	-0.53	0.37
L) RAM correct in first 8	Estimate	Std. Error	Lower CI	Upper CI
(Intercept)	1.53	0.51	0.51	2.54
Bee size	0.01	0.09	-0.16	0.18
M) RAM , correct before revisit	Estimate	Std. Error	Lower CI	Upper CI
Treatment (5 ppb)	0.55	0.56	-0.54	1.64
Treatment (10 ppb)	0.25	0.37	-0.48	0.98
Treatment (250 ppb)	0.49	0.52	-0.52	1.51
Bee size	0.00	0.19	-0.38	0.38
