# Supplementary material for: No evidence for negative impacts of acute sulfoxaflor exposure on bee olfactory conditioning or working memory
Source: PeerJ. 2019 Aug 12;7:e7208. doi: 10.7717/peerj.7208 (PMC6694785; doi:10.7717/peerj.7208)
Supplement: Supplemental Information 4 — Supplementary test: Does sulfoxaflor influence bee behaviour? [file peerj-07-7208-s006.docx]

**No evidence for negative impacts of acute sulfoxaflor exposure on bee olfactory conditioning or working memory**

Harry Siviter^*†1^, Alfie Scott^†1^, [Grégoire](javascript:;) Pasquier^1^, Christopher D Pull^1^, Mark J F Brown^1^ & Ellouise Leadbeater^1^

^1^School of Biological Sciences, Royal Holloway University of London, Egham, Surrey, TW20 0EX, UK

^†^These authors contributed equally

* Corresponding author:

Harry Siviter

School of Biological Sciences

Royal Holloway University of London

Egham, Surrey, TW20 0EX, UK

Harry.Siviter.2016@live.rhul.ac.uk

**Impacts of sulfoxaflor exposure on bee behaviour**

As in Lämsä *et al.* (2018) the time it took the bumblebees to start flying once in the arena was used as a proxy of foraging motivation. Time to start flying was analysed used a linear mixed effect model, with treatment, bee size and their interaction included as fixed factors and covariates respectably. Colony was included as a random factor. There was one outlier in the 250ppb treatment (See Figure S1), which was removed, to improve model fit. The dependant variable was also square rooted to improve model fit.

As a proxy for foraging speed we analysed the time to drink from the first landing platform. As above, we analysed this using a linear mixed effect model, with treatment, bee size, their interaction as fixed factors and covariates respectably with colony included as a random factor. The dependant variable was also square rooted to improve model fit.

**Results**

We found no effect of sulfoxaflor exposure on time to start flying (Figure S1; lmer, 5ppb treatment PE = -0.24, 95% CI = -1.96 to 1.34; 10ppb PE = 0.24, 95% CI = -1.42 to 2.05; 250ppb PE = 1.16, 95% CI = -0.19 to 3.16) or foraging speed (Figure S2; lmer, 5ppb treatment PE = -2.40, 95% CI = -15.55 to 10.74; 10ppb PE = -3.37, 95% CI = -20.03 to 13.28; 250ppb PE = -3.18, 95% CI = -19.19 to 12.81).

**Figure S1: Bumblebee performance on the radial arm maze: Time (seconds) that is took bees to drink from the first flower.**

**Figure S2: Bumblebee performance on the radial arm maze: Time (seconds) that is took bees to drink once they started flying.**
